# Hybridization of surface lattice modes: towards plasmonic metasurfaces with high flexible tunability

**DOI:** 10.1515/nanoph-2023-0121

**Published:** 2023-04-24

**Authors:** Macilia Braïk, Théo Geronimi-Jourdain, Stéphanie Lau-Truong, Abderrahmane Belkhir, Sarra Gam-Derouich, Alexandre Chevillot-Biraud, Claire Mangeney, Nordin Félidj

**Affiliations:** ITODYS, CNRS, Université Paris Cité, F-75006 Paris, France; LPCQ, Université Mouloud Mammeri, BP 17 RP, 15000 Tizi-Ouzou, Algeria; LCBPT, CNRS, Université Paris Cité, F-75006 Paris, France

**Keywords:** hybridization, plasmonics, surface lattice modes

## Abstract

When assembled in periodic arrangements, metallic nanostructures (NSs) support plasmonic surface lattice (SL) resonances resulting from long-range interactions these surface lattice resonances differ radically from localized surface plasmon (LSP). Similarly to the hybridization of LSP resonances, observed in short-range interactions, we demonstrate the possibility to generate a hybridization of surface lattice (SL) plasmon resonances, by the excitation of grazing order diffraction within the metasurface. This hybridization leads to the emergence of *bonding* and *anti-bonding* modes. If hybridization of LSP modes has been widely described in recent literature, there is still no experimental proof-of-concept reporting such hybridization with SL plasmon resonances. We fill this gap in the present paper by considering surfaces made of binary arrays with unit cells made of two gold disks of distinct diameters. We demonstrate the possibility to maximize or cancel the interaction between the hybridized SL resonances by simply controlling the distance between particles. All our experimental results are supported by FDTD calculations. The hybridization of SL modes results in much richer hybridization scenario in terms of wavelength and quality factor control, compared to a hybridization of LSP in a short-range configuration. It offers unprecedented opportunities to generate innovative optical devices, with high flexible tunability.

## 1 Introduction

Confining light at the nanoscale is at the origin of many applications in nano-optics, including the design of efficient optoelectronic devices, molecular sensors, and surface enhanced substrates orphotocatalysts [1–3]. Metal nanostructures (NSs) are particularly remarkable for concentrating the incident light, due to the excitation of localized surface plasmon (LSP) resonances [4]. These LSPs modes correspond to a collective oscillation of conduction electrons at the metallic NS surface, excited by an electromagnetic wave [5]. The LSP modes result in an enhanced extinction in the far field, and a strong confinement of the electromagnetic field at the NSs surface [6]. The LSP wavelength depends on many parameters, among them, the size and shape of the NSs, the nature of the metal (mainly gold and silver, particularly efficient in the visible and near-infrared spectral ranges), or the surrounding medium [7, 8].

Hybridization of plasmonic modes is an interesting strategy in order to extend the range of possibilities in terms of optical response, and to develop narrow resonances [9]. So far, the usual way to generate hybridized LSP modes is to consider short-range interactions, for instance within a dimer of NSs [10–12]. It has been shown that breaking the symmetry of the base element by designing NSs of distinct sizes, leads to hybrid LSP modes, radiating in the far-field [13]. The very first work by A. D. Humphrey et al. mentioned the emergence of such modes, through the design of dimers of silver disks with two different diameters, resulting in a symmetrical mode with non-zero dipole moments and an anti-symmetrical one with weak dipole moment, so-called gray mode [14]. However, one critical issue in short-range interactions is the difficulty in controlling the distance between the two NSs within a dimer, preventing a deep study of hybridization in plasmonics [15]. Indeed, the gap distance needs to be as small as possible (typically below 5 nm), in order to observe such hybridized modes, which is rather difficult to monitor [16].

A recent work by S.-D. Liu et al. showed that the short-range interaction of LSP resonances of trimers composed of distinct gold NSs diamaters, also led to two hybrid LSP modes, characterized by two broad extinction bands, as illustrated by a diagram of energy similar to that of molecular orbitals [13]. Interestingly, the coupling of these hybrid LSP modes and the Rayleigh anomalies of the array, led to an additional sharp resonance assigned as the formation of a surface lattice (SL) modes such SL modes, originating from coherent scattering due to the periodicity of the NSs array, stimulate a growing interest since a few years, as they offer unprecedented opportunities to tune the optical properties, and to yield resonances with high quality factors [17–21].

In recent theoretical works, hybrization of SL resonances was clearly demonstrated [22, 23]. The authors found that binary NS arrays can support SL resonances resulting in very high quality factors, depending on the position of the two NSs of distinct sizes within the unit cell. However, the hybridization of SL resonances has never been observed experimentally so far. In our work, we fill this gap and propose a simple way to reveal experimentally the hybridization of SL resonances, without considering any short-range interactions. To meet this challenge, we designed binary arrays made of gold disks of two distinct diameters, using electron-beam lithography, deposited on an indium tin oxide (ITO) coated glass substrate. In line with previous theoretical demonstration, we show herein experimentally that the hybridization of SL resonances can be described as the electromagnetic analog of the molecular orbital theory, with the emergence of bonding and anti-bonding modes, similarly to hybrid LSP observed in short-range interactions. Remarkably, we show that the hybridization of SL resonances offer new means to manipulate the optical response of plasmonic nanostructures, such as the resonances wavelength and their quality factor, in contrast to the possibilities offered by hybrid LSP modes. We also show that the *anti-bonding* mode can be cancelled, by simply changing the interparticle distance [24, 25]. Breaking the symmetry offers us a much richer hybridization scenario, compared to a hybridization of LSP in short-range configuration, in terms of wavelengths and quality factors control, and therefore, is very promising for the design of plasmonic platforms for instance in the context of molecular sensing [20, 26], [27], [28], [29].

## 2 Results and discussion

**Figure 1:**
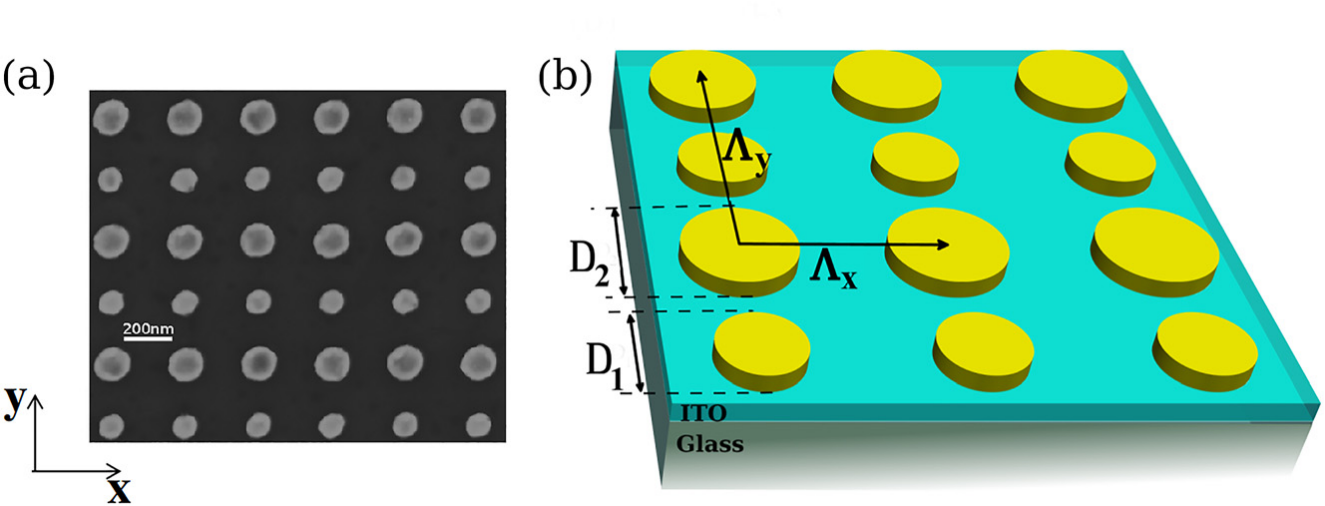
Description of the samples (a) SEM image of a typical binary array of gold disks. The diameters of the disks are *D*_1_ = 100 nm and *D*_2_ = 150 nm. The grating constants are Λ_*X*_ = 300 nm, and Λ_*Y*_ = 500 nm along *X* and *Y*, respectively. The height of the disks is fixed to *h* = 50 nm. They are deposited on an ITO layer (80 nm thick) supported by a glass substrate with refractive index *n* = 1.5; (b) schematic of a binary array of gold disks of diameters *D*_1_ and *D*_2_.

In order to observe a hybridization of SL modes, we considered regular arrays of gold nanostructures, made by electron beam lithography (EBL). This technique provides the possibility to properly monitor the shape of NPs and array parameters, and consequently their optical properties. The description of the procedure of fabrication is detailed in the section *Methods*. We considered arrays of gold nanodisks of two different diameters *D*_1_ = 100 nm and *D*_2_ = 150 nm. The height of the disks has been fixed at *h* = 50 nm. Figure 1 shows a typical example of scanning electron microscopy (SEM) image (Figure 1(a)), and its scheme (Figure 1(a)). In this figure, the grating constant was set at Λ_*X*_ = 300 nm along the *X* axis, and Λ_*Y*_ = 500 nm along the *Y* axis. The grating constant represents the distance between two consecutive nanodisks of same diameters, along the *X* and *Y* directions, respectively. The disks have been deposited on an ITO layer (80 nm thick) supported by a glass substrate with a refractive index *n* = 1.5. All the spectra were recorded in air as the superstrate, since many applications in nano-optics require working in asymmetric media [30].

The far-field optical response of the binary array of gold disks with grating constants Λ_*X*_ = 300 nm and Λ_*Y*_ = 300 nm (array A), were probed by a UV-visible extinction micro-spectrometer using a microscope objective ×10, with a selected numerical aperture (N.A.) of 0.25. The N.A. has been chosen in order to compensate the high dispersivity of SL resonances. Indeed, for a higher N.A., the light collected from surface lattice resonances may result in a broadening of the bands. The grating constants along *X* and *Y* axes for array A have been chosen such that no short-range interactions take place. The incident polarization was fixed parallel to the *X* axis.

**Figure 2:**
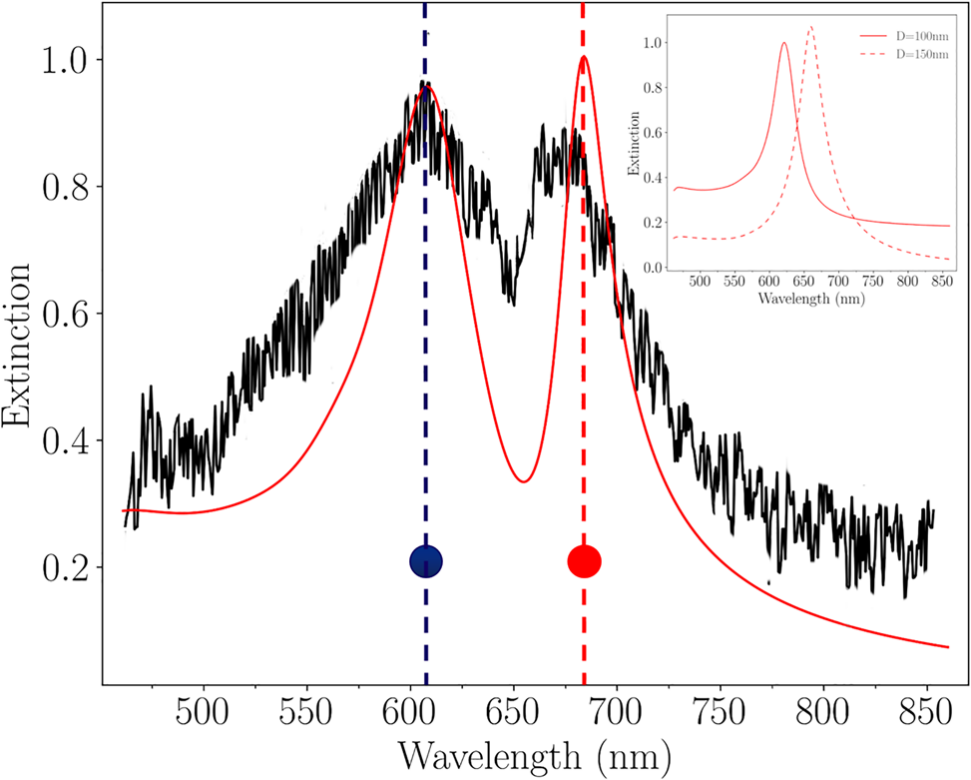
Experimental (in black) and calculated (in red) extinction spectra recorded in air, at normal incidence for a polarization along the *X* axis, for a binary array of disks of diameters (*D*_1_; *D*_2_) = (100 nm; 150 nm) (array A); (in insert) calculated extinction spectra in air recorded at normal incidence for a polarization along the *X* axis for an array of gold disks of diameter *D*_1_ = 100 nm (array B, in solid line), and for disks of diameter *D*_2_ = 150 nm (array C, in dashed line). For all the arrays, the grating constant is fixed to Λ_*X*_ = Λ_*Y*_ = 300 nm.

Figure 2 displays the experimental and calculated extinction spectra of the array A. The calculated spectrum has been modeled by the finite difference time domain (FDTD) method. Its description is detailed in the section *Methods*. Two distinct maxima of extinction are clearly evidenced, located at $\lambda_{blue}^{A}$ = 608 nm (for the blue-shifted surface plasmon band), and $\lambda_{red}^{A}$ = 684 nm (for the red-shifted surface plasmon band). A very good agreement between the calculated and experimental spectra is obtained. This double resonance is assigned to a direct hybridization of surface plasmon resonances, as discussed in the next paragraphs. In comparison, we also displayed, in the insert of Figure 2, the calculated extinction spectra of two distinct square arrays composed of one-size disks with diameters *D*_1_ = 100 nm (array B), and *D*_2_ = 150 nm (array C), respectively. The grating constant has been fixed at Λ= 300 nm, and the height of the disks at *h* = 50 nm for arrays B and C. The two modes exhibited by the binary array A are not simply the superposition of the two distinct plasmon modes of dipolar nature, observed from individual disks for arrays B *λ*_*B*_ = 622 nm, and for array C at *λ*_*C*_ = 656 nm (insert in Figure 2). The wavelengths of the two maxima observed for array A, located at $\lambda_{blue}^{A}$ = 608 nm and $\lambda_{red}^{A}$ = 684 nm, suggests the emergence, in array A, of two hybrid surface plasmon (HSP) modes.

At this point, it is important to point out that the disks are far away from each other, and thus not coupled in short-range distances. The interparticle distances chosen in our studies are such that long-range interactions are present, as confirmed by calculations through the FDTD method (Figure SI 1(a) and (b)). Figure SI 1(a) displays the extinction spectra of arrays of disks of 100 nm of diameters, and Figure SI 1(b), the extinction spectra of arrays of disks of 150 nm diameters, for distinct grating constants Λ_*y*_ varying from 270 to 500 nm (the grating constant is fixed at Λ_*x*_ = 300 nm). The plasmon bands are red-shifted for grating constants equal or higher than Λ_*y*_ = 250 nm (compared to non-coupled NSs), indicating a long-range interaction, associated to the Rayleigh anomaly of (0,1) order (even if this interaction is not at its maximum). It means that the extinction bands observed in the far-field, at the chosen grating constants, are due to the excitation of SLR modes, before considering any hybridization of SLRs. Note that the regime of non-coupled NSs (in short and long-range interactions), associated to LSP excitation, takes place for grating constants located around Λ_*y*_ = 200 nm, for both arrays with disks of diameter *D*_1_ = 100 nm and *D*_2_ = 150 nm, as shown in Figure SI 2. In this figure, displaying the plasmon band wavelength versus the grating constant, the regime of non-coupled NSs is evidenced by the absence of red-shift, consecutive to a short- or long-range interaction. Note that the use of the term hybridization here is in reference to hybridization of two SLRs, as illustrated in Figure SI 3. Figure SI 3 shows that the initial arrays with disks of same diameters (Figure SI 3(a) and (b)) exhibit a SLR strongly coupled to the Rayleigh anomaly at long grating constant (Λ_*y*_ = 420 nm). The optical response of the binary array (Figure SI 3(c)) displays two plasmon resonances, resulting from the interaction between the SLRs of the disks of different diameters. The two distinct SLR modes can interact, and generates hybrid SLR modes. In other words, the hybridization between SLR modes of the two disks of distinct diameters at long distance is made possible by the delocalized nature of these modes. The delocalized nature is evidenced in Figure SI 4, showing the normalized intensity of the electric field distribution (calculated by the FDTD method), at the resonance wavelengths, in the plan above the discs arrays of diameter *D*_1_ = 100 nm (a) and *D*_2_ = 150 nm (b). The grating constants are fixed at Λ_*x*_ = 300 nm and Λ_*y*_ = 420 nm. The fact that the propagation of a photonic mode is observed (evidenced by horizontal maxima in between the NSs), cleary evidences the delocalized nature of the plasmon mode (its wavelength is near the maximum of the extinction band). The two distinct SLR modes can thus interact, and generates hybrid SLR modes, like the observation of an overlap on short-range LSPRs, leading to hybrid modes. In other words, the hybridization between SLR modes of the two disks of distinct diameters at long distance is made possible by the delocalized nature of these modes.

**Figure 3:**
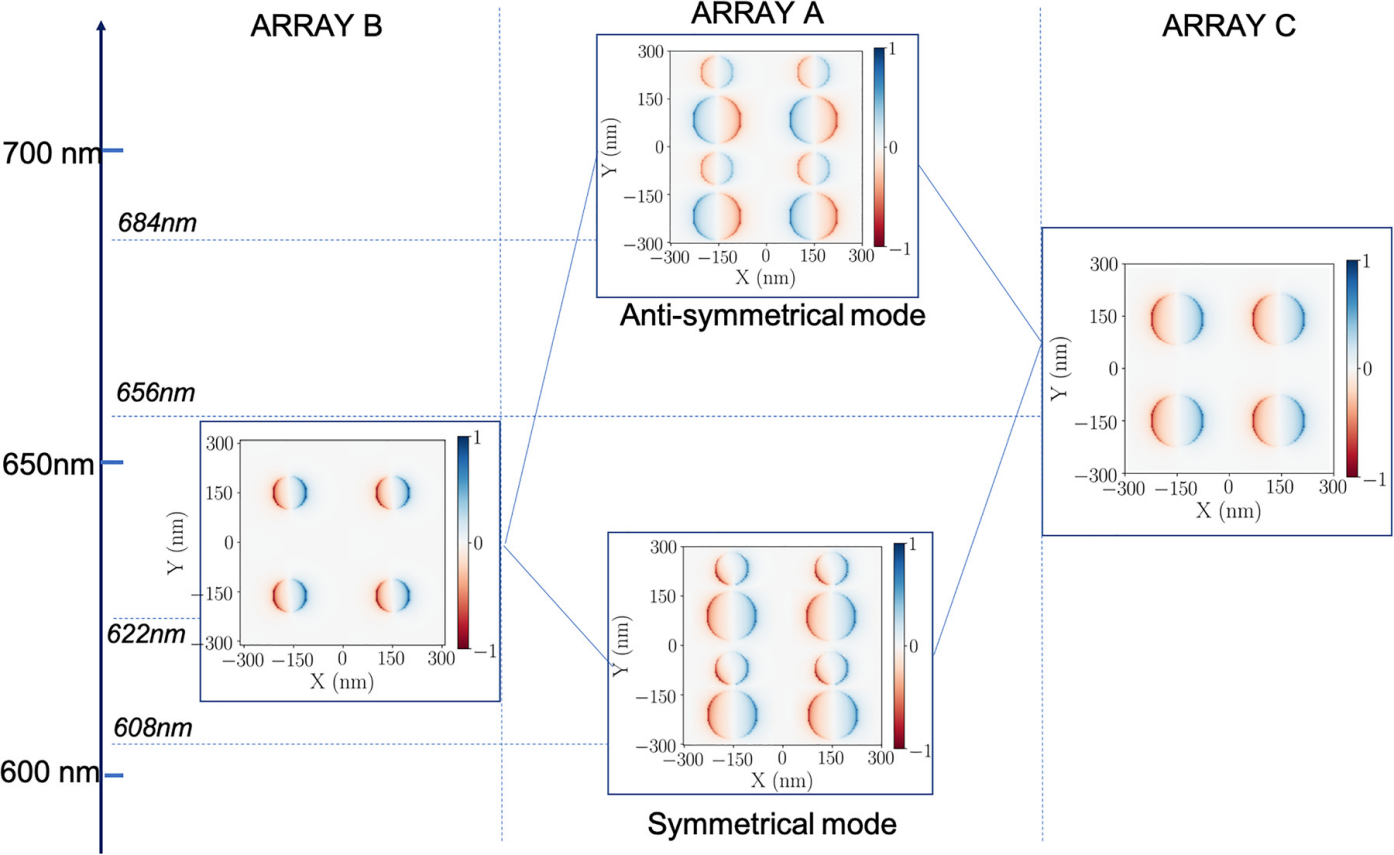
Diagram of the plasmonic hybridization displaying, in the frames, the real part of the normal component of the electric field calculated at $\lambda_{blue}^{A}$ and $\lambda_{red}^{A}$ for the array A, and at *λ*_*B*_ and *λ*_*C*_ for arrays B and C, respectively. The colors indicate the sign of the field, and give the charge distribution (negative in blue and positive in red). All the distributions are calculated at 3 nm above the disks at the selected resonance wavelengths indicated in the panel of Figure 2. The polarization direction is along the *X* axis.

To establish the nature of these two modes, we considered the oscillating dipole moments associated to these modes. We plotted, in Figure 3, the charge distribution of the disks of diameters *D*_1_ and *D*_2_ at the wavelengths $\lambda_{blue}^{A}$ and $\lambda_{red}^{A}$ (for the array A), as well as the charge distribution for array B (with disks of diameter *D*_1_ = 100 nm), and array C (with disks of diameter *D*_2_ = 150 nm). From the charge distributions (Figure 3), it can be concluded that: (i) the plasmon mode at $\lambda_{blue}^{A}$ corresponds to dipoles oscillating in phase for the disks of diameters *D*_1_ and *D*_2_, designated as a symmetrical mode; (ii) the plasmon mode at $\lambda_{red}^{A}$ is such that there is a *π* phase difference in the oscillations between the disks of diameters *D*_1_ and *D*_2_, designed as an anti-symmetrical mode. The appearance of the two HSP modes is due to the interaction between the SL resonances of the two discs of different diameters, enabled by a grating effect (Rayleigh anomaly). The anti-symmetrical mode results in a dipole moment which is almost zero because of out-of phase dipoles between the disks of diameters *D*_1_ and *D*_2_, as expected. As an interesting consequence, its spectral linewidth is narrower, as confirmed in Figure 2. Such hybrid plasmon mode can thus store more electromagnetic energy, compared to LSP modes with a strong dipolar character.

To some extent, the optical response of the array A can be compared to that of closely spaced pairs of particles of different diameters interacting through a near-field coupling, recently investigated by A. D. Humphrey et al. [14]. The authors evidenced, by extinction spectroscopy, two hybridized plasmon modes due to a short-range coupling, with an anti-symmetrical mode becoming radiative due to the difference in diameters within the dimers [14]. They evidenced in this way, two localized surface plasmon resonances, named “gray” modes. In our case, it is shown that there is no need to induce a short distance coupling, generally difficult to achieve and monitor in terms of plasmon wavelengths, in order to generate a hybridization of surface plasmon modes, in contrast to the work of A. D. Humphrey et al. In another study by W. Zhao et al., the authors mentioned the emergence of a sharp Fano resonance through lattice coupling effects, within a binary array with particles of alternating diameters along the *X* and *Y* axes (a different configuration from ours) [31]. The reflection spectra revealed two modes corresponding to two equivalent arrays oscillating in opposite phase leading to a dip (dark mode) in the spectrum. In our specific configuration, different from the cited one, the origin of the two maxima is assigned to two hybridized modes, or “gray” plasmonic modes, arising from constructive and destructive interferences (as described in Figure 3). The two bands, associated to charge oscillation in phase for the symmetrical mode, and to charge oscillation out of phase for the anti-symmetrical one, result from the delocalized nature of the SLR modes. These two hybrid modes are thus associated to SLR modes. Interestingly, due to the delocalized nature of these SLR modes, there is no need to be in a short-range regime in order to observe them. The delocalized nature of the SLR makes possible these interferences between the disks of distinct diameters. Due to the breaking of symmetry, both hybrid modes re-emit in the far-field at distinct wavelengths, due to a resulting non-zero dipolar moment in both cases.

**Figure 4:**
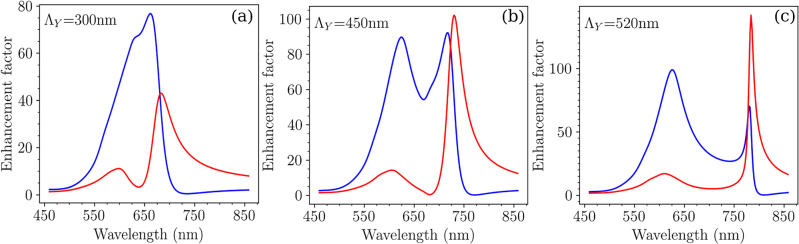
Spectral profile of the average intensity of the local electric field in the vicinity of the disks of diameters *D*_1_ = 100 nm (in blue) and *D*_2_ = 150 nm (in red) for distinct grating constants along the *Y* axis: 300 nm (a), 450 nm (b), and 520 nm (c). The grating constant along the *X* axis remains constant at 300 nm. The incident polarization is set along the *X* axis.

One important aspect is the impact of the near-field spectral response resulting from such hybridization. The spectral dependence of the average intensity of the local electric field around both disks, calculated by the FDTD method, is displayed in Figure 4(a) for the array A. Interestingly, for the symmetrical mode at $\lambda_{blue}^{A}$ , the maximum of intensity of the electric field is mainly located around the small disks of diameter *D*_1_. This suggests that the symmetrical HSP mode observed in the extinction spectrum at $\lambda_{blue}^{A}$ is mainly due to the contribution of the small disks of diameter *D*_1_ (see Figure 2). In the case of the asymmetrical HSP mode at $\lambda_{red}^{A}$ , both disks (*D*_1_, *D*_2_) display significant intensities of the electric field, but still a little bit higher for the smaller disks.

**Figure 5:**
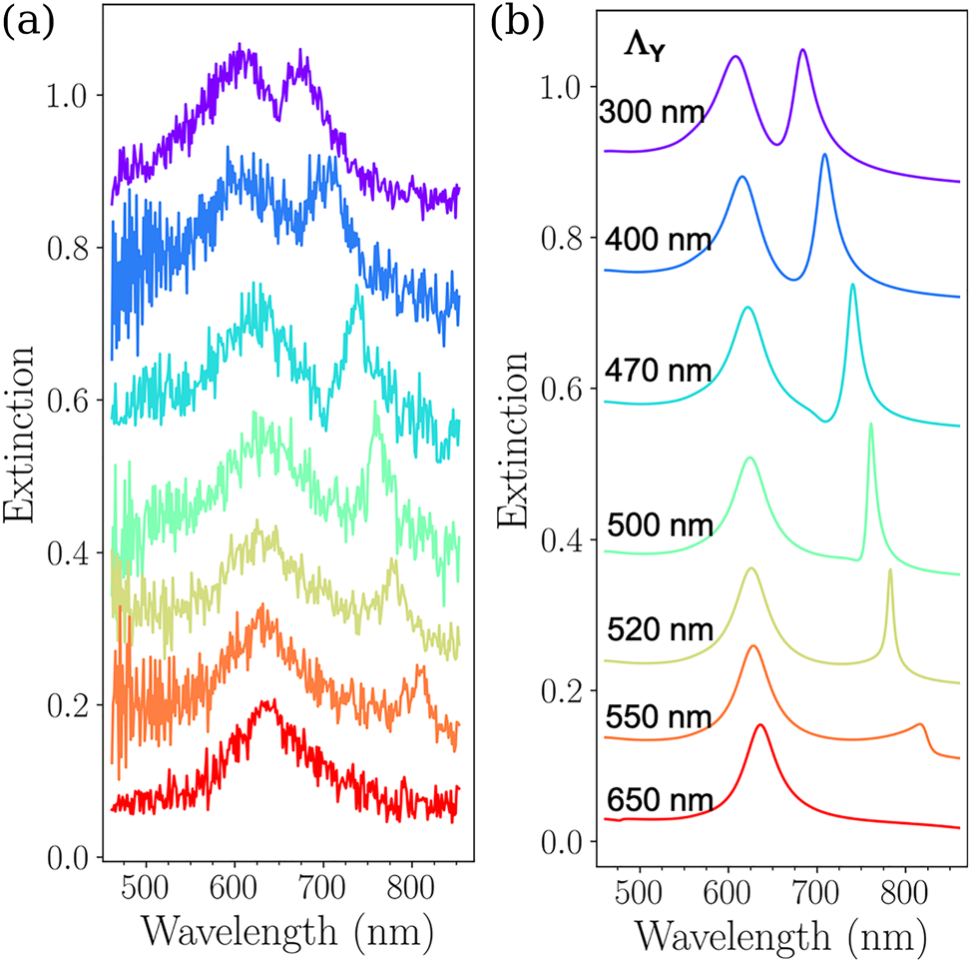
Far-field optical properties (a) Experimental and (b) calculated extinction spectra (by the FDTD method) of the binary arrays of gold disks with diameters (*D*_1_; *D*_2_) = (100 nm; 150 nm), deposited on ITO coated glass substrates. The extinction spectra are recorded in air at normal incidence, for an incident polarization along the *X* axis. The grating constant Λ_*X*_ is fixed to 300 nm and Λ_*Y*_ varies from 300 to 650 nm. For sake of clarity, the spectra have been vertically offset.

The impact of the grating constant on the wavelength of the two hybridized modes is an important question. By changing the grating constant, one is expected to impact significantly the HSP features, through long-range interactions. In the following, we demonstrate that we can precisely monitor the wavelength of these modes, and in particular, improve the quality factor of the asymmetric mode, with enhanced values compared to those of classical regular arrays of plasmonic nanostructures, which is of main importance in the context of the design of enhanced chemical sensors, or high-Q optical devices [24, 32], [33], [34]. We fabricated binary arrays with grating constants varying from Λ_*Y*_ = 300 nm–650 nm. The grating constant along the *X* axis has been fixed at Λ_*X*_ = 300 nm. The diameters and heights of the disks remained the same (with (*D*_1_; *D*_2_) = (100 nm; 150 nm)). The polarization direction was set along the *X* axis. For such polarization, long-range interactions take place perpendicularly to the incident polarization, along the *Y* axis [35]. Figure 5(a) and (b) display the experimental and calculated extinction spectra of the binary arrays of gold nanodisks. The calculated spectra nicely match the experimental ones for all the grating constants considered.

**Figure 6:**
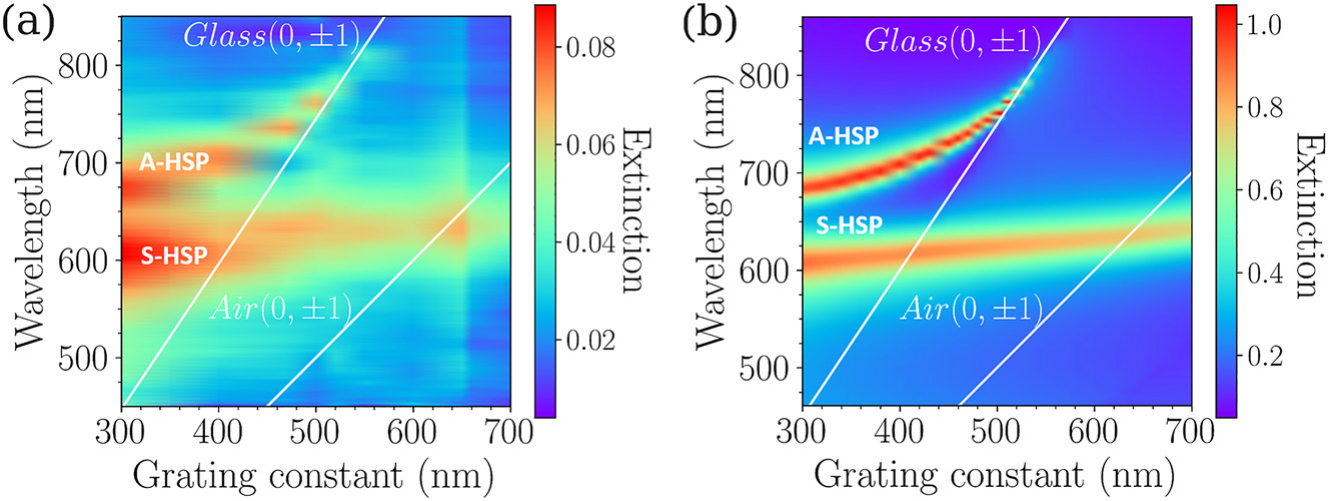
Impact of the grating constant (a) Experimental and (b) calculated dispersion diagrams of the extinction spectra (using the FDTD method) of the binary arrays: wavelengths of the symmetrical (S-HSP) and asymmetrical (A-HSP) modes versus the grating constant Λ_*Y*_. The polarization direction is along the *X* axis. The Rayleigh anomaly position is displayed in white lines for the (0,±1) orders in the air and the (0,±1) orders in the substrate.

Except for the array of grating constant Λ_*Y*_ = 650 nm, all the extinction spectra reveal two maxima attributed to the two HSP modes, as illustrated in Figure 3. However, some significant differences between the two HSP modes can be pointed out: the maximum at $\lambda_{blue}^{A}$ is slightly red-shifted when Λ_*Y*_ increases. This behavior is even more evident in Figure 6(a) and (b), displaying the experimental and calculated dispersion diagrams of the binary arrays, respectively. Both experimental and calculated diagrams are in very good qualitative agreement: the wavelength of the symmetrical HSP mode is poorly sensitive to the increase of the grating constant, crossing the Rayleigh anomaly position corresponding to the (0,±1) orders in the substrate, while the asymmetrical HSP mode at $\lambda_{red}^{A}$ red-shifts when the grating constant increases. In addition, the wavelength of this mode converges towards the position of the Rayleigh anomaly for the (0,±1) orders in the substrate, with a decreasing FWHM, minimum for Λ_*Y*_ = 520 nm (Figure 5(a) and (b)). This mode is thus strongly affected by the grating constant along the *Y* axis.

It is noteworthy that, if the calculated and experimental dispersion diagrams are in very good qualitative agreement, the experimental one evidences a larger FWHM compared to the calculated one. This difference can be attributed to non-perfect round-shaped disks (including some surface roughness, not taken into account in the calculations) [36], a slight dispersion in diameter from one disk to the other, but also to a N.A. which is too high, and collects a large range of angles, leading to a broadening of the observed modes.

**Figure 7:**
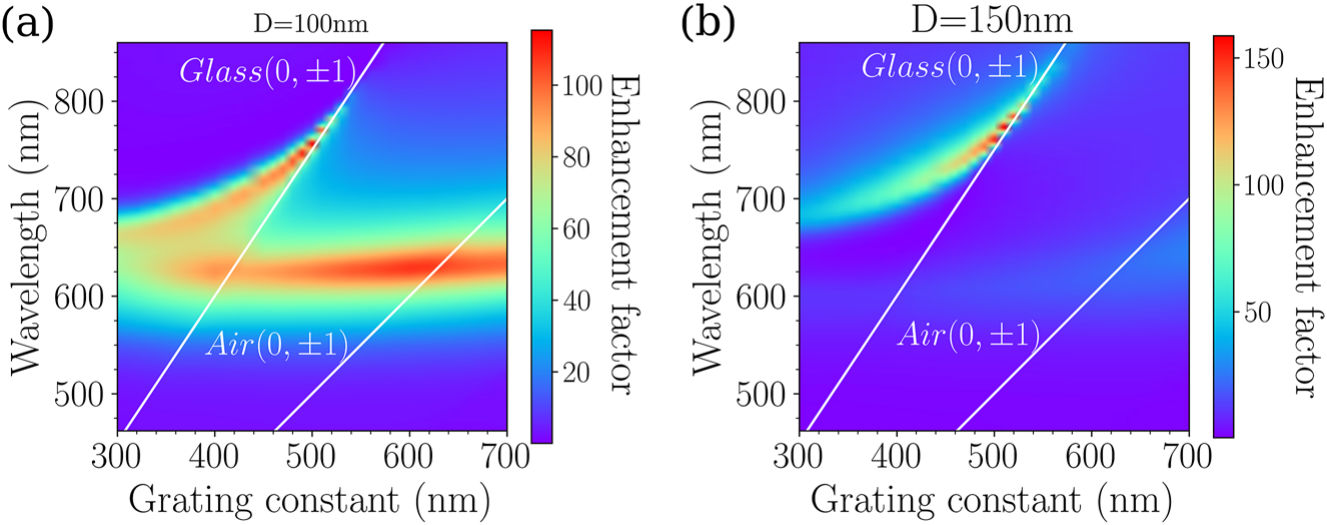
Calculated dispersion diagrams of the average intensity of the electric field on the binary arrays in the vicinity of the disks of diameter *D*_1_ = 100 nm (a), and in the vicinity of the disks of diameter *D*_2_ = 150 nm (b). The incident polarization is set along the *X* axis.

**Figure 8:**
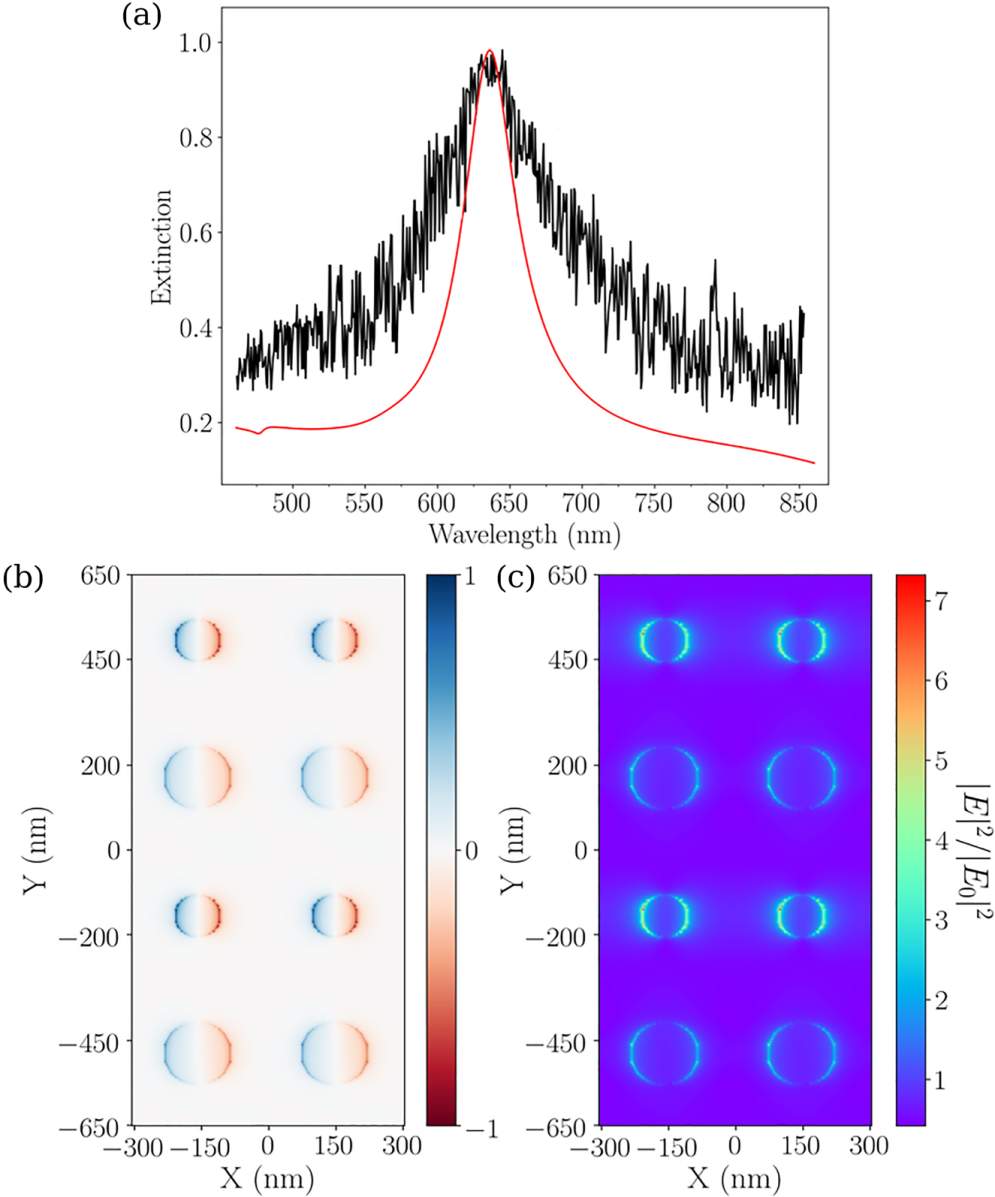
Optical response (a) Experimental and calculated extinction spectra of the binary array of gold disks with a grating constant of 650 nm along the *Y* axis (300 nm along the *X* axis); (b) mapping of the charge distribution at *λ* = 637 nm; (c) mapping of the intensity of the electric field at *λ* = 637 nm (polarization is along the *X* axis).

In the context of surface enhanced spectroscopies, it is of main importance to probe the impact of the grating constant of such binary arrays on their near-field optical response. Figure 7(a) and (b) display the dispersion diagrams of the electric field enhancement (average electric field intensity) of the binary arrays in the vicinity of the disks of diameter *D*_1_ = 100 nm, and of the disks of diameter *D*_2_ = 150 nm. The incident polarization remains along the *X* axis. For the disks of diameter *D*_1_ = 100 nm (Figure 7(a)), the average electric field intensity versus the grating constant displays 2 branches. The first branch at $\lambda_{blue}^{A}$ is such that the intensity is poorly affected by the grating constant. The second branch at $\lambda_{red}^{A}$ is red-shifted and follows the (0,±1) orders due to the substrate. To quantify more precisely the spatial location of the confined energy around each disk, the spectral dependence of the intensities are given in Figure 4(b) and (c): for the following grating constants: Λ_*Y*_ = 450 nm (b), and 520 nm (c) (Λ_*X*_ is maintained at 300 nm). From these calculations, one can conclude that: (i) the average intensity is quite significant in a broad spectral range, whatever the grating constant, and mostly located around the disks of diameter *D*_1_ = 100 nm; (ii) in the case of the disks of diameter *D*_2_ = 150 nm (Figure 7(b)), the average intensity versus the grating constant mainly reveals one maximum corresponding to $\lambda_{red}^{A}$ , and its spectral dependence follows the position of the Rayleigh anomaly for the (0,±1) orders in the glass; (iii): the average intensity, at *λ*_red_ is of the same magnitude for both disk diameters, for a grating constant of Λ_*X*_ = 450 nm (Figure 4(b)). For a grating constant of Λ_*X*_ = 520 nm, the intensity is higher in the vicinity of the disks of diameters *D*_2_ = 150 nm, than those of diameters *D*_1_ = 100 nm (Figure 4(c)). We can thus control the location of maximum of energy around the disks by tuning the grating constant along the *Y* axis (Figure 8).

As mentioned before, one intriguing result is the absence of the asymmetrical HSP mode in the extinction spectrum, related to the grating constant Λ_*Y*_ = 650 nm (Figure 5(a)). The absence of such mode is confirmed by the FDTD calculation (Figure 5(b)). In order to explain this absence in the spectrum, FDTD calculations of the charge distribution and the intensity of the local electric field have been considered at *λ* = 637 nm, associated to the maximum of extinction in the spectrum (see Figure 8(a)). We interpret the presence of a single extinction band as follows: the critical grating constant, corresponding to the maximum of excitation of the asymmetrical HSP mode, is observed at Λ_*Y*_ = 550 nm. For grating constants Λ_*Y*_ above 550 nm, the asymmetrical HSP mode is thus expected to be less efficient (broadened and even blue-shifted), but does not appear in the conditions our experiments (the spectra are recorded in air), as shown in Figure 8(a). For grating constants Λ_*Y*_ above 550 nm, the asymmetrical HSP mode is thus expected to be less efficient (broadened, and even blue-shifted), but does not appear in the conditions our experiments (the spectra are recorded in air), as shown in Figure 8(a). It is shown in Figure 8(b), that the charge oscillations correspond to dipoles oscillating in phase for the disks of *D*_1_ = 100 nm and *D*_2_ = 150 nm, and thus, only the symmetrical mode remains in the extinction spectrum. In addition, these charge distribution are more evidenced on the disks of diameters *D*_1_ = 100 nm, corresponding to a much higher intensity of the electric field in the vicinity of those disks (Figure 8(c)). In conclusion, we are facing a case where the main optical contribution raises from the disks of diameters *D*_1_ = 100 nm and the largest disks do not resonate (or poorly). This example evidences the richness of our strategy, for which a hybridization of surface plasmon lattice resonances can lead to a total inhibition of the asymmetrical hybridized mode by adapting the adequate grating constant.

**Figure 9:**
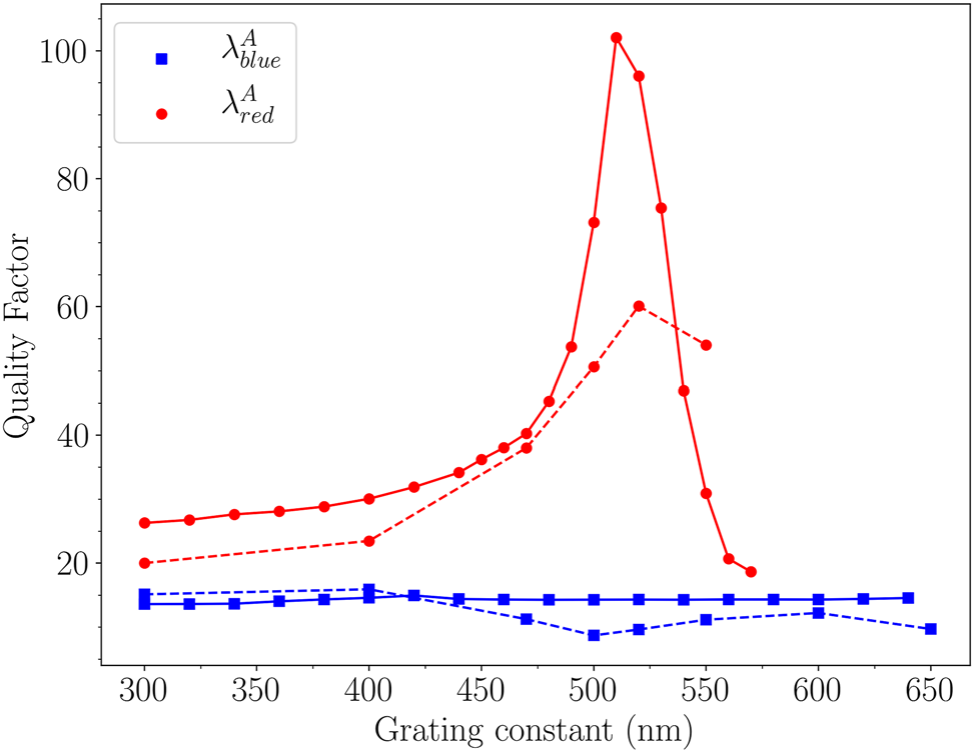
Quality factor of the two HPS modes versus the grating constant, deduced from the experimental (dashed line) and calculated (solid line) extinction spectra of the binary arrays of gold disks of Figure 5(a) and (b): for the symmetrical HSP mode (in blue), and for the asymmetrical HSP mode (in red).

Finally, the optical performances of the binary arrays were evaluated in the context of molecular sensing or high-Q devices. The main parameter is related to the quality factor *Q* defined as *Q* = *E*_max_/(Δ*E*_1/2_), where *E*_max_ is the energy at the maximum of one HSP band and Δ*E*_1/2_, its FWHM. Figure 9 displays the Q-factor of the two HSP modes versus the grating constant, deduced from the experimental and calculated extinction spectra of the binary arrays of gold disks from Figure 5(a) and (b). Both experimental (dashed line) and calculated (solid line) Q-factors are in good qualitative agreements. From the values of the Q-factors versus the grating constant, we can conclude that: (i) for the symmetrical HSP mode (indicated in blue in Figure 9), the Q-factor (≃10) is not influenced by the grating constant. This invariance was expected since the corresponding extinction spectra have similar FWHM, whatever the grating constant; (ii) for the asymmetrical HSP mode (indicated in red in Figure 9), the reduced linewidth of the asymmetrical HSP modes leads to much higher values of *Q*, compared to the ones of the symmetrical HSP modes. In addition, when the grating constant increases, the experimental values *Q*_exp_ also increase reaching a maximum at 60 for a grating constant of Λ_*Y*_ = 520 nm, corresponding to a maximum of long-range interaction. For such grating constant, in the asymmetrical case, the intensity of the local electric field is enhanced in the vicinity of the two disks of both diameters, giving rise to a much larger useful surface resulting in a greater stored energy. However, it was found that *Q*_th_ is almost twice higher than *Q*_exp_ (≃1.7 × *Q*_exp_). This difference between the experimental and theoretical Q-factors is due to the fact that the FWHM is strongly affected by the nanoscale surface roughness (NSR) of the disks, due to the evaporation process of the EBL fabrication. An annealing of the arrays would lead to considerably decrease this NSR, due to a decrease of the imaginary part of the dielectric constant, as already described [36–38]. In our FDTD calculations, the NSR is hardly to be considered and thus has not been taken into account, which explains this difference in Q-factors between the experimental and the calculated data.

The quality factors we found using the binary arrays can be compared to regular arrays constituted of disks of identical diameters. We found, in a recent work, that the maximum of *Q* = 25 was obtained, in a strong long-range interaction regime, in air and in normal incidence [39]. Its value is more than twice smaller than those obtained for the binary arrays presented in this work. More generally, the Q-factors we found are of the same order of magnitude, compared from recent literature, for gold NSs, in normal incidence and in an asymmetrical medium [40]. Although the improvement of *Q* is not within the main scope of the paper, it would be of great interest, in the context of the applications cited above, to proceed to an annealing of the NSs, and consider their optical responses in a homogeneous surrounding medium, in order to optimize even more the quality factors.

## 3 Conclusions

We presented a detailed study of the optical properties of binary arrays of gold disks with distinct diameters. We evidenced the emergence of two hybrid plasmonic modes, through the excitation of grazing higher order diffraction. The symmetrical mode is associated to oscillating charges in phase between the disks of two distinct diameters, while the anti-symmetrical mode is assigned to a π phase difference in the charge oscillations, between the disks of two distinct diameters. There is no need of short-range interaction to observe the emergence of both modes. Indeed, taking advantage of long-range interactions, allows to precisely monitor their spectral features, in terms of resonance wavelength and quality factor. In such regime, the quality factor can be significantly enhanced with experimental values reaching *Q* = 60. All our experimental results are in very good agreement with FDTD calculations. We believe that the engineering of such substrates is particularly promising in the context of molecular sensing, surface enhanced spectroscopies, and more generally, high Q-factors optical devices.

## Supplementary Material

Supplementary Material Details
