# Supplementary material for: Hybridization of surface lattice modes: towards plasmonic metasurfaces with high flexible tunability
Source: Nanophotonics. 2023 Apr 24;12(12):2179–88. doi: 10.1515/nanoph-2023-0121 (PMC11501960; doi:10.1515/nanoph-2023-0121)
Supplement: Supplementary file 1 — Supplementary Material Details [file j_nanoph-2023-0121_suppl.docx]

**Hybridization of surface lattice modes: towards plasmonic meta-surfaces with high flexible tunability**

Macilia Braïk, Théo Geronimi Jourdain, Stéphanie Lau-Truong, Abderrahmane Belkhir, Sarra Gam-Derouich, Alexandre Chevillot-Biraud, Claire Mangeney, and Nordin Félidj*

**Macilia Braïk, Théo Geronimi-Jourdain, Stéphanie Lau-Truong, Sarra Gam-Derouich, Alexandre Chevillot-Biraud, Nordin Félidj***, Université Paris Cité, ITODYS, CNRS, F-75006 Paris, France

**Macilia Braïk, Abderrahmane Belkhir,** Université Mouloud Mammeri, LPCQ, BP 17 RP, 15000 Tizi-Ouzou, Algeria

**Claire Mangeney**, Université Paris Cité, LCBPT, CNRS, F-75006 Paris, France

*Nordin Félidj : corresponding author


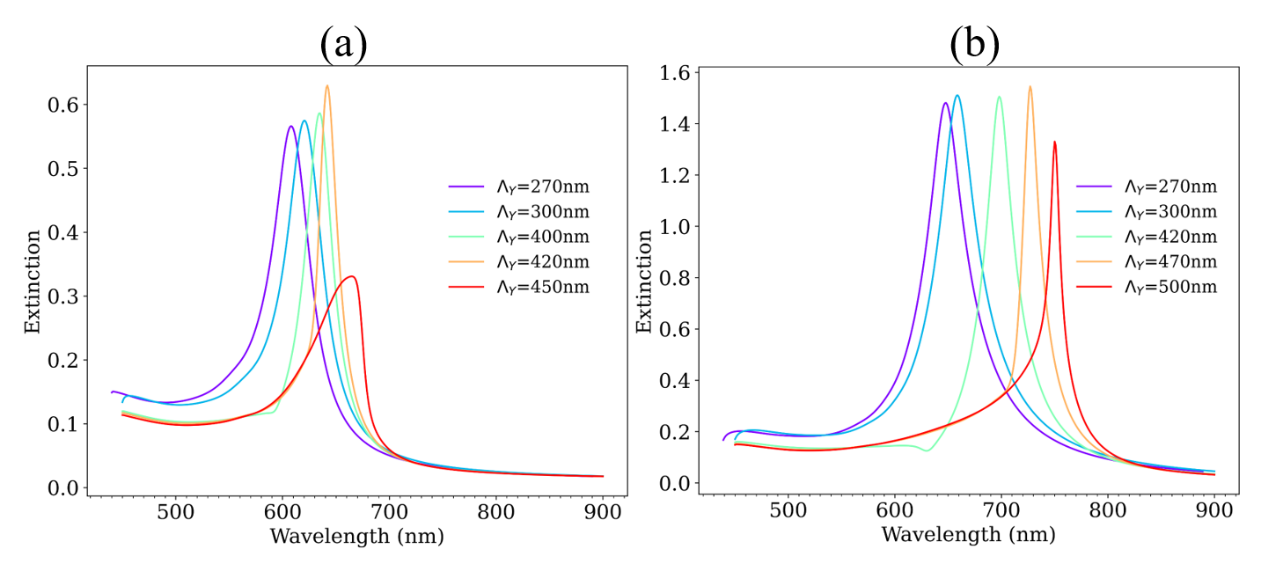


Figure SI1 Calculated extinction spectra of arrays of gold disks with diameters D_1_=100 nm in (a) and D_2_=150 nm in (b), deposited on ITO coated glass substrates. The extinction spectra are recorded in air at normal incidence, for an incident polarization along the X axis. The grating constant is fixed to Λ_x_ = 300 nm and Λ_y_ varies from 270 to 500 nm.


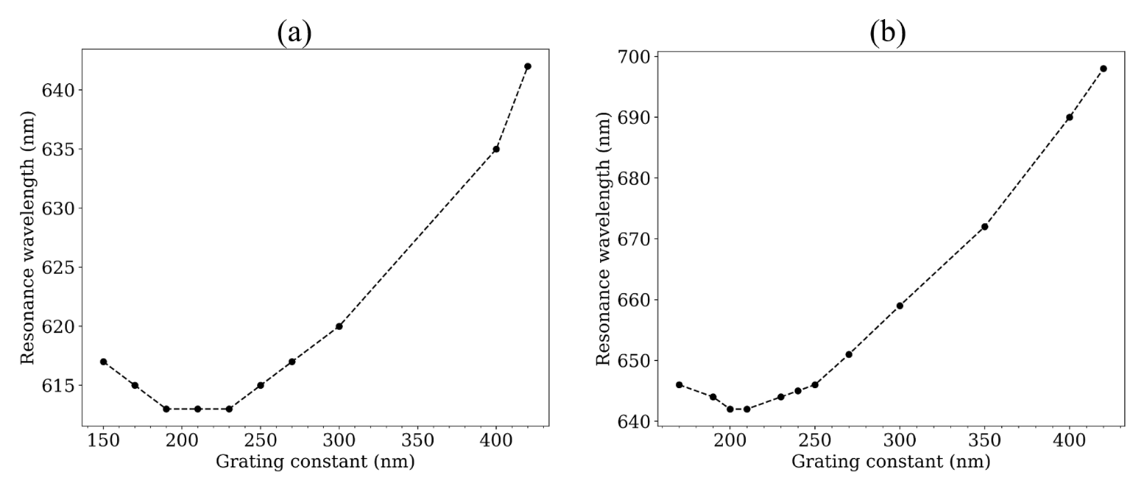


Figure SI2 Spectral position of the resonance deduced from the calculated extinction spectra of the discs array of diameter D_1_ = 100 nm in (a) and D_2_ = 150 nm in (b). The grating constant is fixed to Λ_x_ = 300 nm and Λ_y_ varies from 270 to 500 nm.


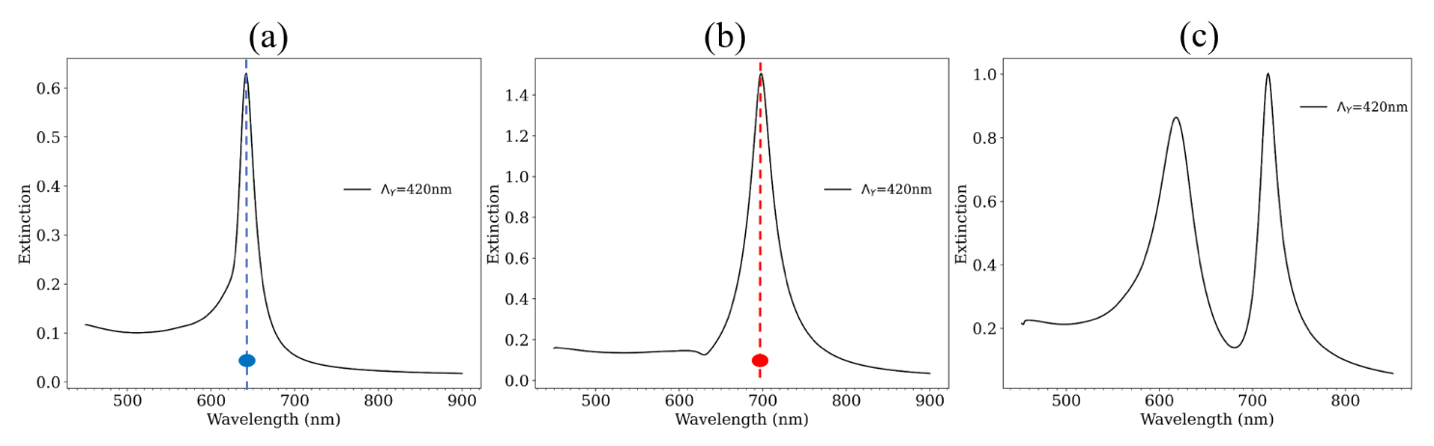


Figure SI3 Calculated extinction spectra in air, at normal incidence for a polarization along the X axis, for (a) array of gold disks of diameter D_1_ = 100 nm, (b) array of gold disks of diameter D_2_ = 150 nm and (c) binary array of disks of diameters (D_1_; D_2_) = (100 nm; 150 nm). For all the arrays, the grating constants are fixed to Λ_x_= 300 nm and Λ_y_ = 420 nm.


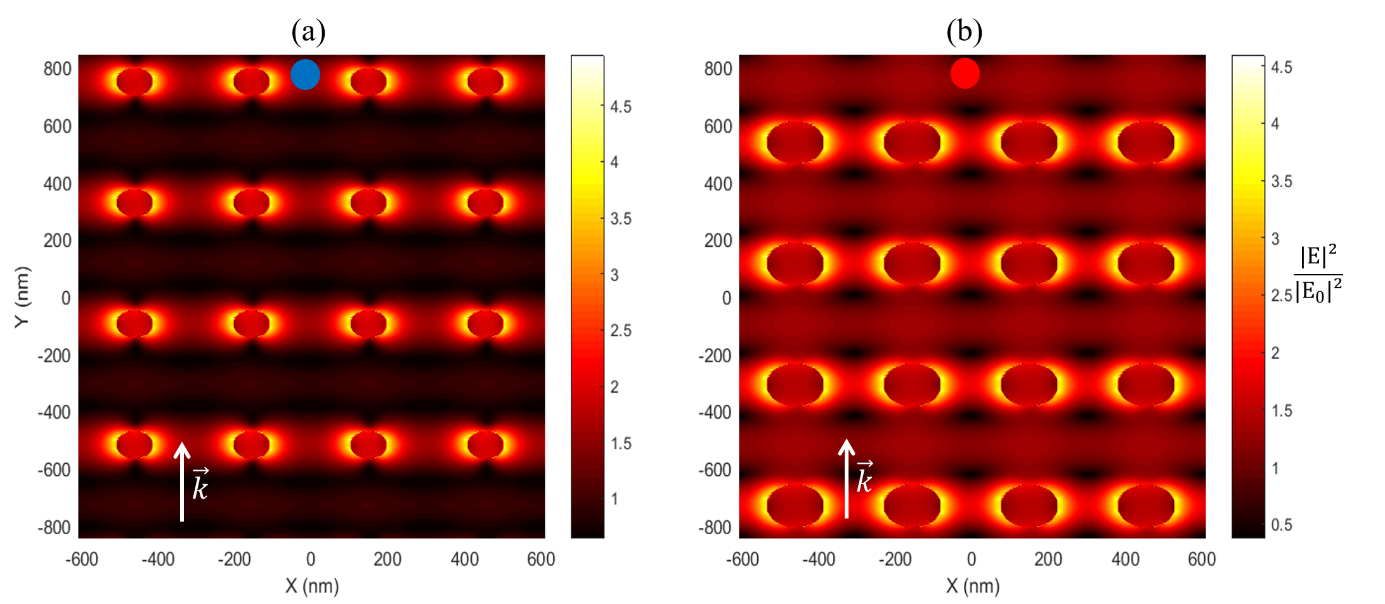


Figure SI4: Normalized intensity of the electric field distribution (calculated by the FDTD method), at the resonance wavelength, in the plan above the discs arrays of diameter D_1_ = 100 nm in (a) and D_2_= 150 nm in (b). The grating constants still fixed at Λ_x_= 300 nm and Λ_y_ = 420 nm. For a better contrast, the values given in *colorbar* represent the normalized intensity power a third.
